# Occupational Disease as the Bane of Workers’ Lives: A Study of Its Incidence in Slovakia. Part 2

**DOI:** 10.3390/ijerph182412990

**Published:** 2021-12-09

**Authors:** Miriam Andrejiova, Miriama Pinosova, Miroslav Badida

**Affiliations:** 1 Department of Applied Mathematics and Informatics, Technical University of Košice, 040 01 Košice, Slovakia; miriam.andrejiova@tuke.sk; 2 Department of Environmental Engineering, Technical University of Košice, 040 01 Košice, Slovakia; miroslav.badida@tuke.sk

**Keywords:** occupational diseases, physical factors, cluster analysis, Slovakia

## Abstract

The main objective of this article is to monitor the development of the number of occupational diseases related to selected physical factors in the working environment (noise, vibration and dust). Each region of Slovakia has its own specific social and economic conditions. Due to the existence of a strong correlation between the several regional variables observed, principal component analysis (PCA) was used to determine the new variables. Cluster analysis was used to group regions with similar characteristics. A dendrogram was created using the average linkage method, which illustrated the similarity of the regions studied. The value of the cophenetic correlation coefficient (CC = 0.90) confirms the validity of the average linkage method. The result of the cluster analysis is the grouping of the eight regions into five homogenic groups (clusters). An analysis of the data shows that Slovakia’s regional differences significantly influence the incidence of occupational diseases in individual regions. It is shown that, in Slovakia, the development of the number of occupational diseases has seen a favourable trend in the long term.

## 1. Introduction

The overall health of a population that supports itself mainly through manual work is affected by a range of unfavourable workplace risks involving multiple physical factors. Long-standing efforts and the gathering of scientific evidence in the area of health and safety overwhelmingly confirmed what many experts have long assumed: that risk factors in the workplace can contribute to health problems that were previously considered to be unrelated to work.

The development of occupational diseases was monitored and evaluated by a large number of authors [1,2,3,4,5,6,7,8,9]. In their articles, they presented retrospective studies that analysed the structure, causes, occurrence and trends in the development of occupational diseases over a certain period of time in a given country. Carder et al. (2015) [10] published an overview of occupational disease reporting systems in EU (European Union) countries participating in the Modernet consortium. The evaluation of occupational diseases in the EU was studied by Nikolson [11]. The global burden of occupational diseases was covered by Lesley Rushton [12]. Howard (2017) [13], for the journal *Occupational Medicine*, stated that “the occupational health and safety issues facing employers, workers, medical practitioners and researchers in the USA are numerous”. Moyo et al. (2015) [14] state that occupational medicine is a completely new discipline in Africa. In most developing countries around the world, there is generally an acute shortage of doctors and OHS services. Piňosová et al. (2021) [15] compiled a detailed chronological overview of the literature on the development of occupational diseases and the historical development of the ILO.

This article is focused on the analysis of occupational diseases related to physical factors whose long-term action can have harmful health effects. The aim of this article is to analyse occupational diseases in Slovakia over the last 23 years. In particular, the data on reported/recognised diseases (Item 28, Item 29, Item 38, and Items 33–34) were directly related to the exposure to four risk factors: noise; vibration; dust; and long-term, one-sided load were taken into account. These risk factors are the factors with the greatest impact on human health. The article provides a detailed analysis of the incidence of the number of occupational diseases using four basic indicators (gender, age, economic sector and region). The number of diseases reported/declared appears to be largely influenced by regional characteristics and differences. For this reason, an analysis of the regional incidence of the number of occupational diseases is also performed through principal component analysis (PCA) and cluster analysis.

## 2. Some Important Occupational Diseases

Noise, an unwanted factor in the working environment, is one of the most important bionegative factors in the environment of a person living in civilization. Noise-related hearing loss ranks third in the global ranking of occupational diseases and can cause physiological and psychological disorders. Fosbroke (1831) [16,17,18] examined “blacksmith’s deafness”, which he attributed to the long-term exposure to loud noises in blacksmiths’ workshops. Fosbroke J. was the first to point out that the onset of “deafness” due to noise exposure is a gradual rather than a sudden process. Currently, the most common definition is “Noise is unwanted sound” most likely introduced in 1932 by Sabine [19]. Daniel Fink (2019) argued that it was imperative that we reflect on the new definition of noise for the current age, as the phrase “noise is unwanted sound” or “noise is undesired sound” only suggests that noise is merely an inconvenience and ignores the modern knowledge on the subject. Therefore, Fink proposed a new definition of noise: “Noise is unwanted and/or harmful sound” [20]. There is no doubt about the harmful effects of noise on hearing. However, individuals of the same age and gender, who work in the same environment at the same time, do not always have the same results from an audiological hearing examination. There is a large individual variation in sensitivity to noise-caused hearing loss. A number of factors that may affect hearing loss are known, for example race, gender, age [21,22], smoking [23,24], hypersensitivity to noise [25], previous noise exposure [26,27], hypertension [28], synergistic effects [29], and others. An important step in assessing the effects of noise is also proposing measures to reduce it; Moravec et al. (2017, 2021) [30,31]. Employees who are exposed to hand and arm vibrations are also exposed to high noise levels. Pyykkö et al. (1981) [32] was the first to suggest that saw operators, in whom VWF (Vibration White Finger) disease is indicated, may develop greater hearing loss than men without VWF indication. Iki et al. (1986, 1994) [33,34], Bovenzi (2006) [35], House et al. (2010) [36], Pettersson et al. (2014) [37], Turcot et al. (2015) [38], supported the hypothesis that VWF increases the risk of hearing loss in those workers using handheld vibration tools.

Exposure to noise at work may cause irreversible damage to hearing. Occupational noise-induced hearing loss (NIHL) is the most common health problem in the world and is difficult to detect as the effects build up over time. The World Health Organization warns that by 2050, nearly 2.5 billion people worldwide, or 1 in 4 people, will be living with a certain degree of hearing loss [39]. The National Institutes of Health (NIH) reports that between 16% and 24% of workers in the world will suffer hearing damage due to high noise levels. According to the Health and Safety Executive (HSE), about 17,000 workers in the UK suffer from deafness, tinnitus or other ear diseases due to excessive noise at work [40]. Chen et al. (2020) [41] produced an extensive review of noise-related hearing loss among workers, stating that the burden associated with occupational noise varies from 11.2% (South African gold miners) to 58% (American construction workers). Noise prevention programs are an important preventive measure in reducing the morbidity of NIHL among workers.

Exposure to vibration is associated with an unpleasant subjective sensation of discomfort, with general fatigue of the body resulting in reduced attention, slowed and impaired perception, and a decrease in motivation and work performance. Vibrations are a frequent risk in the industry, which we were unable to eliminate over a long period. Raynaud’s phenomenon was first described by the son of a Parisian university professor, Maurice Auguste Gabriel Raynaud (1881, French doctor and writer) in 1862 [42]. At the beginning of the 20th century (1911), the Italian doctor Loriga [43] described for the first time the occurrence of tingling, numbness and whitening of the fingers of the hands in stoneworkers and carvers who used a pneumatic hammer without a handle. It is known that some working tools and devices produce vibrations that, after a certain period of time, cause cumulative traumatic disorders, which eventually lead to conditions such as Vibration White Finger on the hands and feet [44], Raynaud’s disease [45], parasthesia [46,47], carpal tunnel syndrome [48], back and neck pain [49,50], headache and dizziness [51], indigestion, and other problems.

The health conditions caused by vibrations manifest themselves slowly. In the beginning, it usually begins with pain. If exposure to vibration continues, pain may develop into injury or illness. It is estimated that around 400,000 Swedish workers are exposed to vibrations for more than 2 h per working day [52]. According to Britain’s HSE, up to 2 million people are at risk from hand–arm vibration. Eurofound, based on the European Working Conditions Surveys (EWCS-2015), reported that in Europe, 33% of men and 10% of women are regularly exposed to vibration, a total of 48.6 million workers [53,54]. In the U.S. (2016), the incidence rate of accidents and occupational diseases was about 23% (774,900) for transportation, warehousing and utilities workers. All workers in these jobs were most likely exposed to both HTV (hand-transmitted vibration) and WBV (whole body vibration). This is also the case for agriculture, forestry and fisheries (15.25%), construction (0.3%), mining (5.8%), and for 12.5% of the people employed in the manufacturing sector [55].

Dust in the workplace is one of the single biggest issues in health and safety and can be a problem in any industry. In the UK, up to 500 construction workers die every year as a result of exposure to silica dust. The HSE’s chief inspector of construction, Sarah Jardine, said: “Around 100 times as many workers die from illnesses caused or made worse by their work than are actually killed in construction accidents” [56]. In early 2019, Cecil E. Roberts, President of the United Mine Workers of America (UMWA) called for changes to the regulation of silica dust in mines and called for stricter standards for working conditions. The U.S. Department of Labor (DOL) reports that about 2.3 million individuals in the U.S. are exposed to silica at work [57]. Despite the growing demand for alternative energy sources, coal is still an important source of energy worldwide. Beer Ch. et al. (2017) [58] stated that 30% of global energy needs are covered by the coal that produces 41% of the world’s electricity. Based on statistics from the International Energy Agency (IEA) [59], the total world coal production in 2019 was 7.921 Mt, an increase of 12.03% compared to 2009, indicating an increasing demand for coal. Annual consumption stands at 5406.9 Mt.

Ramazzini (1700) [60] described nose and eye irritation in pit sawyers and headaches in lumberjacks and was the first to report the adverse effects of wood dust on health. He went on to describe a peculiar form of asthma in those who processed cotton, flax and hemp. He said the dust he observed in their preparation “causes workers to cough constantly.” Exposure to dust increases the prevalence of respiratory diseases such as extrinsic allergic alveolitis [61,62], asthma symptoms [63,64], chronic bronchitis [65], organic dust toxic syndrome [66], chronic obstructive pulmonary disease [67,68], and the incidence of cancer is increased [69,70].

Musculoskeletal disorders that were shown or believed to be partially caused in the workplace were defined as Work-Related Musculoskeletal Disorders (MSDs) [71]. They affect a large number of people, regardless of gender, and tend to lead to long and serious disabilities. In its report, the Healthy Workplaces Campaign 2020–22, the European Agency for Occupational Health and Safety (EU-OSHA) [72] stated that up to 60% of all workers with a work-related health problem identified MSDs as their most serious problem. The main causes of these disorders are excessive stress and repeated movement. According to a report by the U.S. Bureau of Labor Statistics, musculoskeletal incidence rates represented as much as 30% of all cases of injuries and illnesses, with the incidence found to be 27.2 cases per 10,000 workers [73]. The Global Burden of Disease (2020) study, led by Ciez, showed that around 1.71 billion people worldwide have musculoskeletal problems [74].

The severity of these factors stems from the fact that they usually affect large population groups, and, since the health consequences are not clear immediately after exposure, they are underestimated by the public.

## 3. Materials and Methods

### 3.1. Data Sources

The evaluation of the development of the incidence of occupational diseases in Slovakia in the period 1997–2019 was based on data documented by the National Health information Centre (NHIC), which belongs to the Ministry of Health of the Slovak Republic. The status and tasks of the NHIC are regulated by Act no. 153/2013 on the National Health Information System. As part of its activities, NHIC cooperates with institutions such as the Statistical Office of the Slovak Republic, the Office for Health Care Supervision, the Public Health Authority, the State Institute for Drug Control, the institutes of the Slovak Academy of Sciences, healthcare providers, healthcare professionals and staff organizations, health insurance companies, and medical faculties. At the international level, NHIC cooperates with the World Health Organization (WHO), Organization for Economic Co-operation and Development (OECD) and EUROSTAT.

Data from databases were used for analysis, which can be accessed on the website of the Statistical Office of the Slovak Republic, https://Slovak.statistics.sk/STATdat (accessed on 6 July 2021), DATAcube. Their use and free dissemination are regulated by the Creative Commons Attribution License (cc-by) 4.0.

### 3.2. Slovakia

Slovakia (the Slovak Republic, capital city: Bratislava) is a landlocked country in Central Europe that has been part of the European Union since 2004. Approximately 5.45 million inhabitants live in Slovakia, which has a total area of 49,035 km^2^.

**Figure 1:**
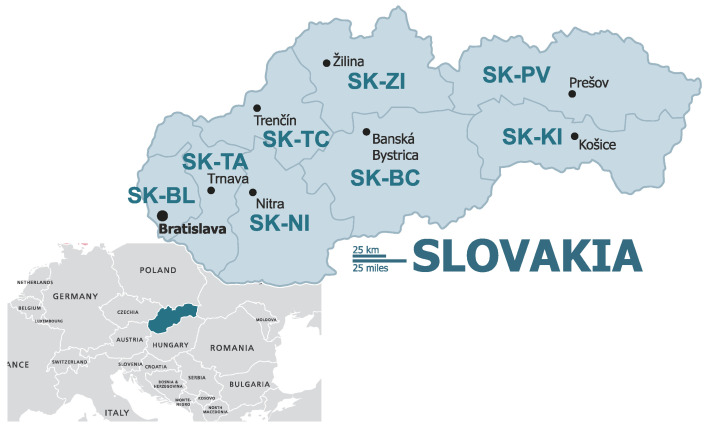
Slovakia and regions of Slovakia (Base https://maproom.net/; © Can Stock Photo Inc./(tele52), accessed on 22 May 2021).

Slovakia currently has 8 regions (Bratislava region SK-BL, Trnava region SK-TA, Nitra region SK-NI, Trenčín region SK-TC, Žilina region SK-ZI, Banská Bystrica region SK-BC, Prešov region SK-PV, and Košice region SK-KI), 79 districts, 140 towns and 2933 municipalities (Figure 1).

The Bratislava region (SK-BL) is the most developed region in Slovakia and differs significantly from other regions. Other regions, particularly those in Eastern Slovakia (Prešov region SK-PV, Košice region SK-KI), lag far behind the Bratislava region.

The Žilina region (SK-ZI) is one of the most industrially oriented regions. The Trnava region (SK-TA) benefits from its geographical proximity to the developing Bratislava region. The Trenčín region (SK-TC) is one of the most industrial regions in Slovakia. The Nitra region (SK-NI) is the most developed agricultural region in terms of agricultural area (more than 74% of the region’s area) and the productivity of local agricultural production. The Banská Bystrica region (SK-BC) is a relatively less developed region, with agricultural and food production mainly concentrated in its southern part.

The Košice region (SK-KI) is a relatively developed region in economic terms with the metallurgical, mechanical, electrotechnical and food industries at the heart of it. The Prešov region (SK-PV) is a less developed region with a long-standing high unemployment rate and significant economic and social disparities compared to other regions.

When monitoring the differences in the regions of Slovakia in terms of the numbers of occupational diseases, we took into account several other input variables: region size, population of the region, average age, working age population, unemployment rate, population working in selected sectors of economic activity (agriculture and forestry, mining and quarrying, industrial production, construction, other sectors).

The values of the selected variables in individual regions of Slovakia for 2020 are shown in Table 1.

### 3.3. Statistical Evaluation Methods

We based the assessment of the incidence of occupational diseases in Slovakia on the period 1997–2019, using the data documented by the National Centre of Health Information (NCHI), which is under the Ministry of Health of the Slovak Republic.

Basic statistical methods were used to analyse the incidence of the number of occupational diseases in Slovakia. In the case of the regional comparison of the development of the number of diseases, principal component analysis (PCA) and cluster analysis were used.

Principal Component Analysis (PCA) is one of the methods of multidimensional analysis. In particular, the aim of the method is to reduce the number of inputs and dependent (correlated) variables with the least possible loss of information. The new latent variables, the principal components, are independent of each other and represent a linear combination of the original variables. Each principal component is characterized by a degree of variability. The first principal component describes the greatest variability of the original values. Other principal components always contribute to variability by a smaller proportion. An adequate number of principal components can be determined using several methods. In practice, the Kaiser-Guttman rule applies, which takes into account all custom values (eigenvalues) greater than 1. Another rule recommends considering only those principal components that explain 70% to 90% of cumulative variance [75].

Cluster analysis is one of the multidimensional statistical methods that examines the similarity of multidimensional structures and the classification of structures into homogeneous groups—or clusters [76,77]. Information on similarity is obtained using different metrics of estimating the distance between two structures (e.g., Euclidian distance). Hierarchical and non-hierarchical methods are distinguished based on the method of obtaining homogeneous groups. Hierarchical clustering methods are based on a gradual grouping of structures, from the most similar to the most varied. There are several methods of hierarchical clustering (e.g., closest neighbour method, furthest neighbour method, average join method, Ward method, etc.). The graphical representation of hierarchical clustering is a tree—a dendrogram. A cophenetic correlation coefficient, CC, may be used to determine the best clustering method. The highest value of the cophenetic correlation coefficient determines the best method of clustering. The closer its value to 1, the more advantageous is the hierarchical cluster method used to express the structure of the analysed data. All of the results of the cluster analysis were obtained using the R package program.

## 4. Results and Discussion

The research focused on selected items (diseases) from the list of occupational diseases in Slovakia that are directly related to physical factors in the working environment, known as (sd-PF). These are one-sided loads on the limbs (Item 29), damage caused by excessive vibration (Item 28), noise (Item 38), or dust (Items 33–34); see also [15]. The aim of the research was to:

- analyse the incidence of recognised selected occupational diseases (1997–2019);
- analyse the incidence of occupational diseases in terms of selected indicators (1997–2019);
- compare the regions of Slovakia in terms of the selected variables.

### 4.1. Incidence of Recognised Selected Occupational Diseases (1997–2019)

**Figure 2:**
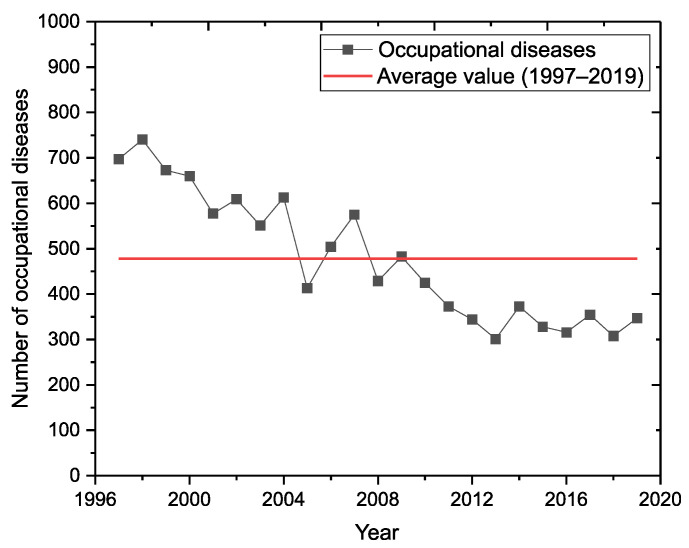
Incidence of all occupational diseases in Slovakia (1997–2019).

According to data from the National Health Information Centre (NHIC), a total of 10,993 newly occurring occupational diseases were reported in Slovakia between 1987 and 2019. The average annual number of cases in the given period was 478 reports. In the long term, we observed a downward trend in the development of reported occupational diseases (Figure 2).

**Figure 3:**
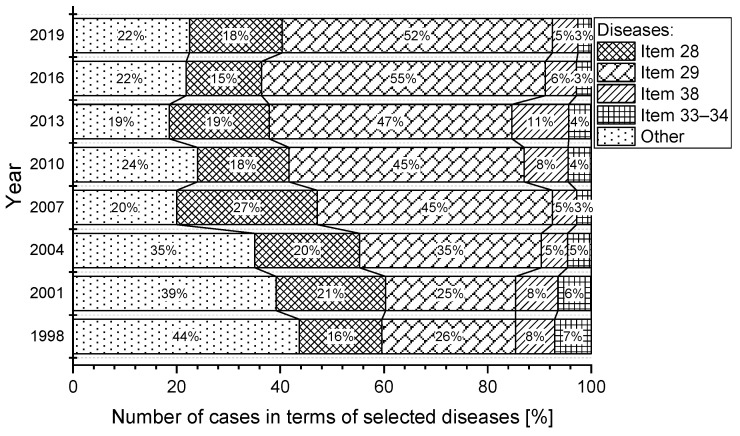
Percentage of selected occupational diseases in Slovakia (1997–2019).

The incidence of occupational diseases in Slovakia for selected items from the list of occupational diseases is shown in Figure 3. Of the total number of occupational diseases recognised, there were 7386 cases in the (sd-PF) category (Item 28, Item 29, Item 38 and Items 33–34), representing almost 67.2% of the total number of registered cases.

**Figure 4:**
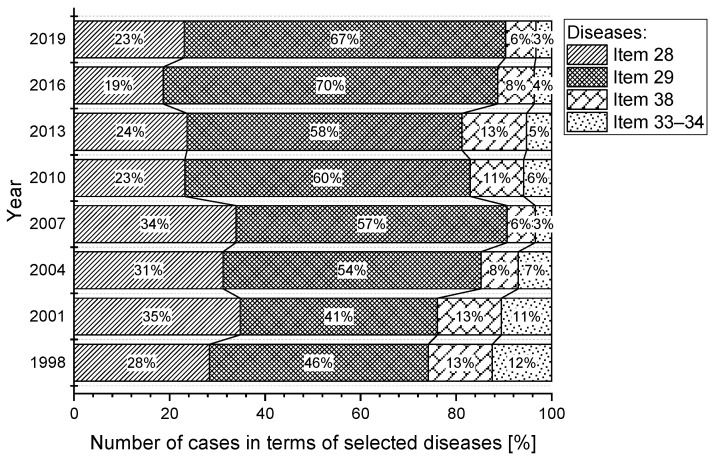
Percentage of number of diseases from the perspective of (sd-PF) (1997–2019).

The most significant percentage of reported cases in the (sd-PF) categories under reference were in Item 29, representing an average of almost 56.3%. In second place is the category, Item 28, with a share of 26.1%. The category, Item 38 has an 11.4% share and the category, Item 33–34, has only a 6.3% share. The percentage of selected occupational diseases in the total number of reported cases in the (sd-PF) categories is shown in Figure 4. For the sake of clarity, only selected years are plotted in the chart.

**Figure 5:**
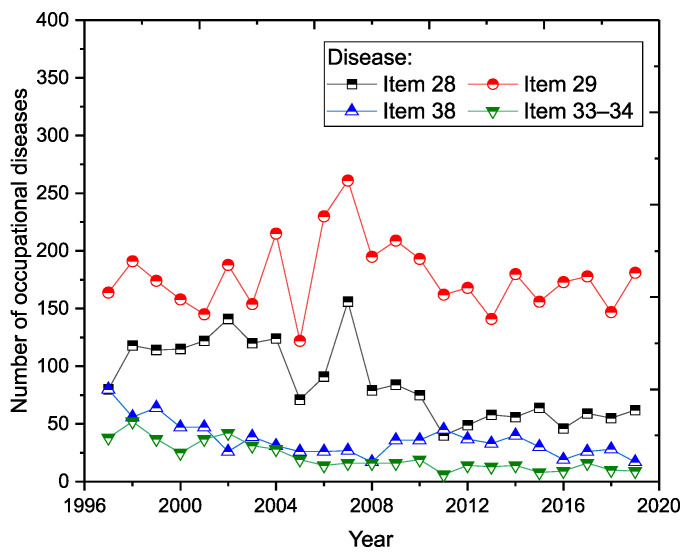
Incidence of occupational diseases from the perspective of (sd-PF) (1997–2019).

In three (sd-PF) categories (Item 28, Item 38, Items 33–34), we see a slightly decreasing trend in the number of reported cases (Figure 5). The most significant decrease is in the category Item 33–34; in 2019, there was almost a 24% decrease in reported cases compared to 1997.

The average annual number of occupational diseases related to limb disorders from long-term, excessive and one-sided load (Item 29) is 178. Despite the decreasing trend in the number of reported new cases (Figure 2), this illness does not have a very positive development (Figure 5). There was a sharp increase in 2006 (230 cases), representing an almost 86% increase compared to 2005. The largest number of reports (261 cases) was recorded in 2007, representing almost 45.4% of the total number of reports in a given year (575) and a 63.5% share in the (sd-PF) category (361). The highest percentage was recorded in 2016, when Item 29 accounted for 70% of all cases in the (sd-PF) category.

The second most common occupational disease is occupational disease from vibrations (Item 28). The average annual number of reported cases is 86. In 2007, there was a significant increase in the number of reported cases (156), with that year representing a 27.1% share of occupational disease in terms of the total number of cases in a given year (575) and almost a 40% share in the selected group of (sd-PF) diseases. Since that year, we observed a significant decrease in the number of reported cases.

Noise-related hearing loss (Item 38) is, on average, in third place in terms of the number of reported cases. The average annual number of reported cases is 36. In the long term, the number of cases reported annually is decreasing. The lowest incidence was recorded in 2008 and 2019; with 17 cases each.

Pneumoconiosis (Items 33–34) [15] has the lowest number of reported cases in each year of the reporting period. The average annual number of reported cases is 24. Since 2002, there has been a significant decrease in the number of reported cases compared to the previous period (1997–2002).

The basic characteristics of the number of reported cases in each (sd-PF) category are given in Table 2.

### 4.2. Incidence of (sd-PF) Occupational Diseases in Terms of Selected Indicators (1997–2019)

For a more detailed analysis of the incidence of (sd-PF) occupational diseases, we selected four indicators (Table 3): the sex of workers (two subcategories), the age category of workers (five subcategories), the sectoral classification of economic activities (five subcategories) and the region of the health establishment where the occupational disease was diagnosed (eight subcategories).

**Figure 6:**
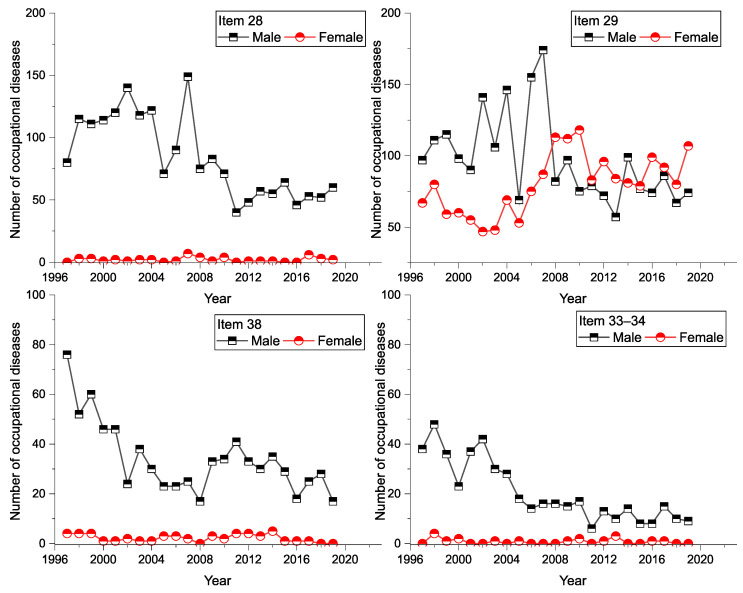
Incidence of (sd-PF) in terms of sex of workers and diseases (1997–2019).

A graphical representation of the incidence of (sd-PF) occupational diseases in terms of the sex of workers is shown in Figure 6. In men, we can see a significantly decreasing trend in the number of occupational diseases admitted for each item on the list of diseases.

It was found that men have a greater share in the total number of (sd-PF) diseases. In the Item 28 category, men have an 84% share, in the category of Items 33–34 they have a 93.6% share. This is also the case with item 29 (94.4% share). An overview of the number of gender-specific diseases is available in Table 4.

The highest incidence of (sd-PF) occupational diseases by workers’ age category in the given period was in the age group from 50 to 59 years (Age4: 3104 cases), representing a 42% share of the total. In second place was the group from 40 to 49 years (Age3: 2871 cases), who represented almost 39% of the total. Item 29 was the most commonly reported disease in both age groups with a comparable number of reports (Age4: 1760 cases, Age3: 1758). The number of occurrences, average values and percentages in each age group and items (diseases) is shown in Table 5.

**Figure 7:**
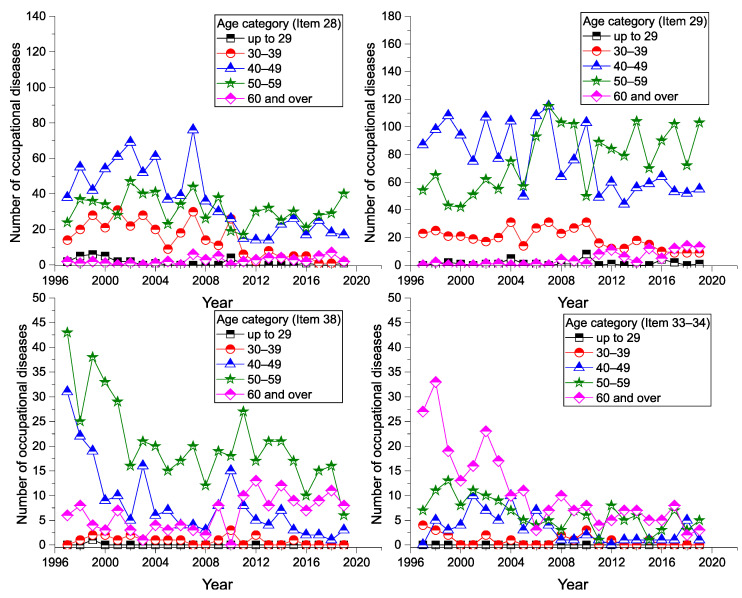
Incidence of (sd-PF) by age of workers and disease (1997–2019).

In recent years, the number of reported cases in the over-60 age group increased slightly, particularly for Item 29. A graphical representation of the incidence of (sd-PF) (Item 28, Item 29, Item 38, and Items 33–34) in the age group view can be seen in (Figure 7).

**Figure 8:**
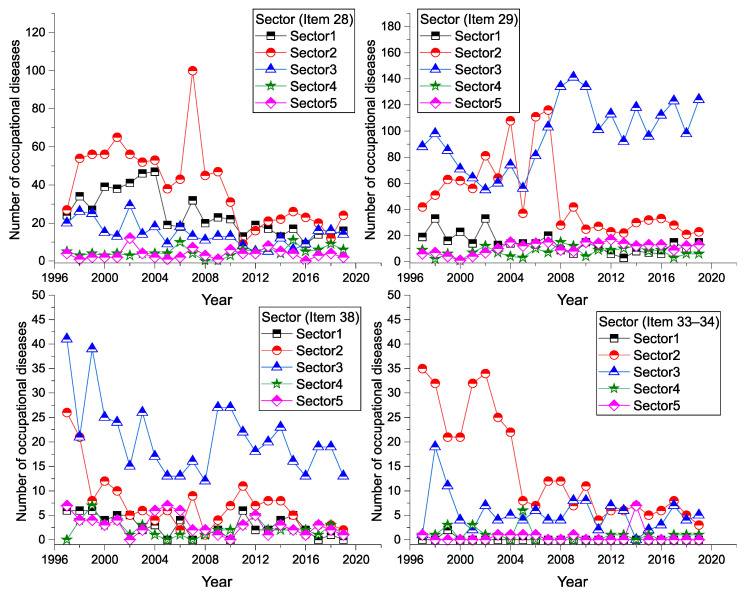
Incidence of (sd-PF) by sectoral economic activity and disease (1997–2019).

A graphical representation of the incidence of diseases, in terms of the sectoral classification of economic activity, is shown in Figure 8. The highest number of reported cases of (sd-PF) by the sectoral classification of economic activities was recorded in the industrial production sector (Sector 3: 3151 cases), representing almost 43% of the total. Mining and quarrying is in second place (Sector 2: 2519 cases), representing a 34% share of the total. The lowest number of reported cases for the period was found in construction (Sector 4: 353 cases, 4.8%).

In the Item 29 category, the number of reported cases has increased sharply in the construction sector since 2007. In other categories of (sd-PF), and other sectors of economic activity, we observe a slightly decreasing trend or a constant trend. The number of occurrences and average values in each sector of economic activity and diseases are shown in Table 6.

There appear to be significant differences in the number of reported cases of occupational diseases in the regions (Table 7). The available data show that for the period from 1997 to 2019, the largest number of reported cases of occupational diseases were in the Košice region (KI: 2479 cases), representing 33.6% of the total number of cases. This is followed by the Banská Bystrica Region (BC: 1527 cases; 20.7%) and the Žilina Region (ZI: 1524 cases; 20.6%). The fewest reported cases were in the Trnava region (TA: 14 cases; 0.2%).

In the Košice region (KI), the number of reported cases in the categories of Item 28, Item 29 and Item 38 is almost constant. A slight decrease is observed in the category of Items 33–34. In the Banská Bystrica region (BC), we have seen a decrease in the number of reported cases for all (sd-PF) over the last 5 years. In the Žilina region (ZI), we see a decrease in reported cases, except for Item 29, for which there was an increase in the number of reported cases, with almost 3 times more cases reported in 2019 than in 1997. Since 2010, we saw an increase in reported cases of Item 29 in the Bratislava region (BL). In this region, 34 cases were reported for Item 29 in 2019, an increase of almost 567% compared to 1997. In the Trnava region (TC) there was a sharp decrease in reported occupational diseases for all monitored items. Even in 2019, not a single case of occupational disease was reported in any (sd-PF). In the Prešov region (PV), a decrease in reported cases is observed; for example, in the category of Items 33–34, only one case was reported for the whole period. The total number and average annual value of the number of reported occupational diseases in each region of Slovakia is shown in Table 7, where the percentage of reported diseases in the given regions is also given in terms of the total number of (sd-PF) cases.

**Figure 9:**
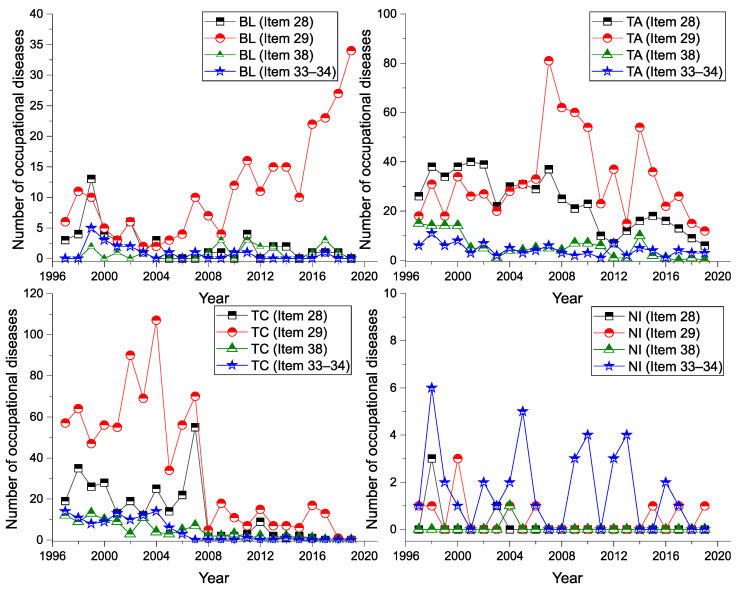
Incidence of (sd-PF) for BL, TA, TC, NI regions and diseases (1997–2019).

**Figure 10:**
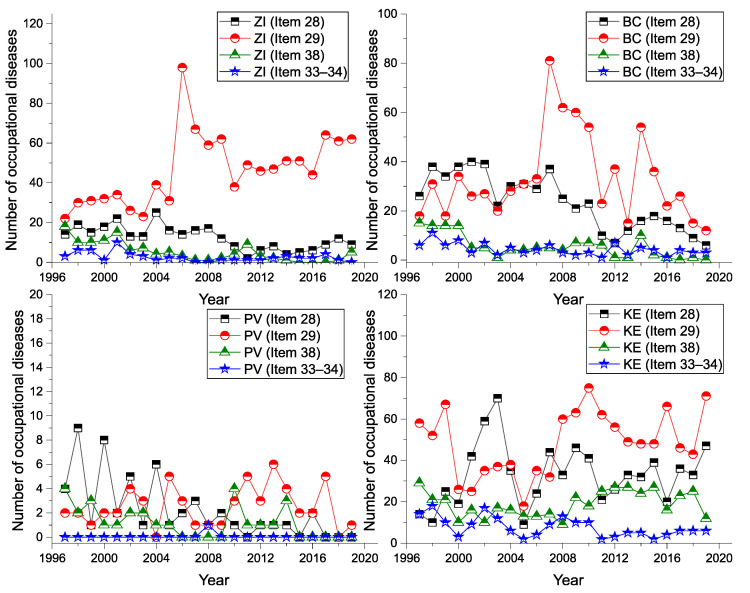
Incidence of (sd-PF) for ZI, BC, PV, KI regions and diseases (1997–2019).

A graphical representation of the incidence of the diseases in each region is shown in Figure 9 and Figure 10.

### 4.3. Comprehensive Comparison of Slovak Regions Using Selected Indicators

For a more comprehensive comparison of regions in terms of the reported number of occupational diseases, we selected further additional data on the regions of Slovakia for the following parameters: region, population of the region, working-age population of the region area, average age of the population of the region, unemployment rate in the region, and the population of the region working in selected sectors of economic activity (Table 8 and Table 9).

In our evaluation, we worked with data representing the average values over the last five years.

Using the regression coefficient, we checked whether the input variables were correlated, i.e., whether there was a correlation between them. Because the variables are measured in different units, a correlation matrix was used to determine dependency (Table 10). The correlation between the two variables is shown in the correlation matrix using the Pearson coefficient of correlation *r*. We used a scale to determine the level (degree) of dependency: no correlation (|r| < 0.29), poor correlation (0.30 < |y| < 0.49), medium correlation (0.50 < |r| < 0.79), and strong correlation (S, 0.80 < |r| < 1). If the correlation coefficient r is positive (or negative), there is a direct (or negative) linear dependency between the variables.

The results from the correlation matrix show that there is a very strong, direct correlation between variables A3 and A10 (r = 0.99), A1 and A5 (r = 0.89), A3 and A9 (r = 0.86), and A9 and A10 (r = 0.87). For example, there is medium correlation between variables A3 and A6 (r = −0.72), A7 and A8 (r = 0.77), and A6 and A10 (r = −0.70). It was found that there are relatively strong correlations between many pairs of variables. For this reason, principal component analysis (PCA) was used to identify new independent variables. Using this method, the original variables were replaced by new, independent variables—the principal components.

**Figure 11:**
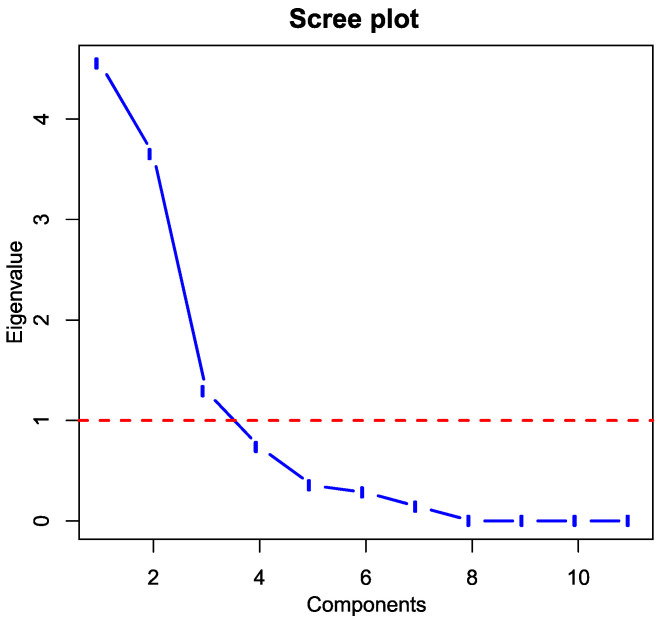
Scree plot (Output: R).

Eigenvalues were used to determine the principal components. The scree plot (Figure 11, blue line) displays eigenvalues for each new independent variable (dimension). The red dotted line corresponds to an eigenvalue of 1. Table 11 shows the eigenvalues and variability of PCA components.

The first dim1 principal component has an eigenvalue of 4.55 (Table 11). The eigenvalue for the second principal component of Dim2 is 3.65, and the third value of Dim3 is 1.29. The results of the PCA method show that the first principal component, Dim1, describes approximately 41.37% of the total data variability; the second component, Dim2, describes 33.17%; and the third component; Dim3, describes 11.72% of total variability.

To determine the appropriate number of principal components, we used the Kaiser-Guttman criterion, according to which all eigenvalues greater than 1 are considered [75]. Subsequently, the first three principal components were used to determine the new variables, which together covered almost 86.3% of the total variability in the data (Table 11).

Table 12 shows the coefficients of the eigenvectors and the component matrix of the coefficients for the first three principal components. The first component of Dim1 consists primarily of variables A1, A2, A3, A5, A6, and A8. The second component of Dim2 consists mostly of the variables A4, A9, and A10. The third principal component of Dim3 consists mostly of the variables A7 and A11.

Cluster analysis, namely hierarchical cluster analysis, was used to group regions of Slovakia with similar characteristics. Euclidian distance was chosen as the distance measure and the first three principal components, Dim1 to Dim3, were selected as input independent variables. The cophenetic correlation coefficient CC (cophenetic correlation coefficient) was used to determine the best clustering method. For the average linkage method, the value of CC = 0.90; for the nearest neighbour method, CC = 0.86; for Ward’s method, CC = 0.75; and, for the median method, CC = 0.87. The highest value of the cophenetic correlation coefficient CC is for the average linkage method, which can be considered the best method of clustering.

**Figure 12:**
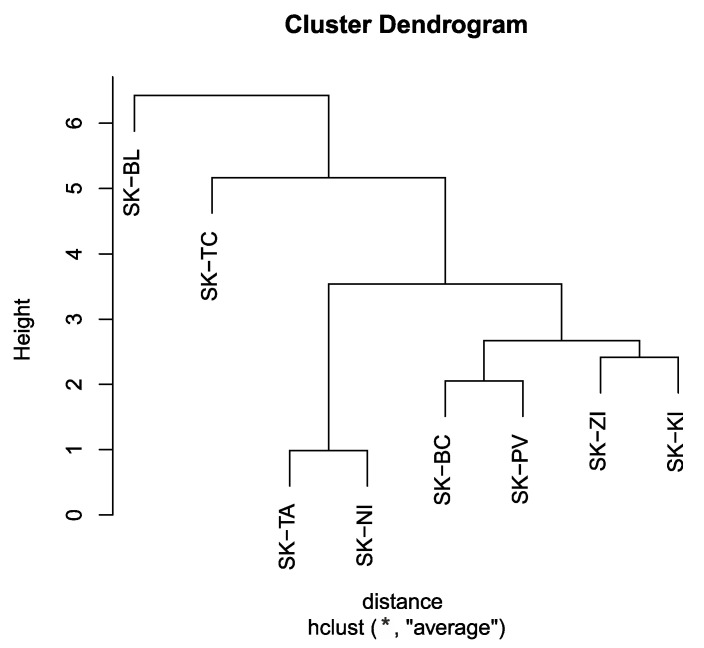
Dendrogram—average linkage method (Output: R).

**Figure 13:**
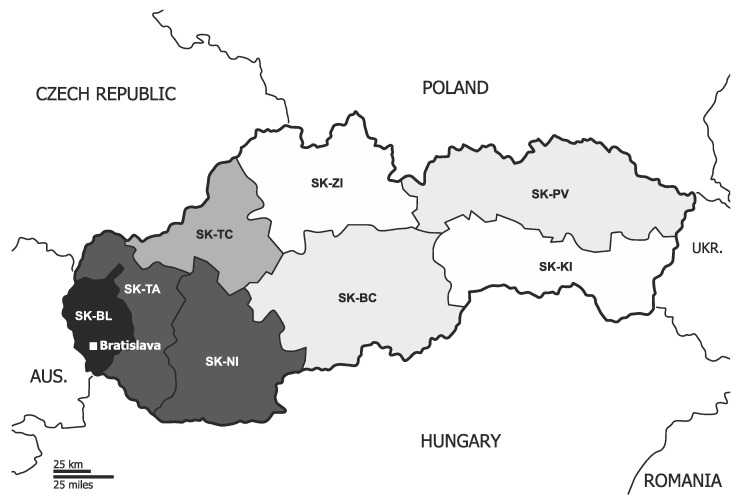
Result of cluster analysis—division of regions (Base © Can Stock Photo Inc./(tele52), accessed on 22 May 2021).

The results of the cluster analysis of selected variables that characterize the regions of Slovakia are shown in Figure 12. From the dendrogram, Slovakia can be divided into five homogeneous clusters based on the selected variables: Cluster 1 (SK-BL), Cluster 2 (SK-TC), Cluster 3 (SK-TA, SK-NI), Cluster 4 (SK-BC, SK-PV) and Cluster 5 (SK-ZI, SK-KI). The regions constituting one cluster are shown in the same colour (Figure 13).

## 5. Conclusions

The results from the analysis of data show that the trend in the number of occupational diseases in Slovakia is generally favourable, i.e., the number of occupational diseases recognised is decreasing in the long term.

It was found that regions can be grouped into five homogeneous groups with similar characteristics. A significantly different group consists of the Bratislava region (SK-BL), where trade and services, as opposed to industrial production, dominate. The Trenčín region (SK-TC) is the most industrial region with a dense transport network. The Košice region (SK-KI) and Žilina region (SK-ZI) are dominated by traditional engineering, metallurgical and electrotechnical industries. In the Banská Bystrica region (SK-BC) and Prešov region (SK-PV), agricultural production plays an important role. The Trnava region (SK-TA) and Nitra region (SK-NI) are characterized by an equal representation of industrial and agricultural production. The analysis of the data shows that the incidence of occupational diseases strongly reflects the regional differences in Slovakia and to some extent is also caused by this difference. It is important to remember that each region has its own specific social and economic conditions, such as the availability of labour, the development of the transport structure and the composition of regional industry, which significantly influences the incidence of occupational diseases in Slovakia.

## Figures and Tables

**Table 1:** Selected characteristics of the regions of Slovakia.

Region	Area (km^2^)	Population (in Thousands)		Average Age (Year)	Unemployment (%)
		Overall	Working Age		
SK-BL	2052.6	673.3	354.5	41.0	3.4
SK-TA	4146.3	565.1	288.4	42.2	5.0
SK-TC	4501.8	583.6	293.5	43.0	3.9
SK-NI	6343.8	676.9	336.0	43.0	5.2
SK-ZI	6808.5	691.3	341.6	40.8	5.5
SK-BB	6454.0	641.2	326.9	42.2	7.9
SK-PV	8972.8	826.6	399.3	39.1	12.1
SK-KI	6754.3	801.8	372.1	39.9	8.8

**Table 2:** Descriptive statistics on the number of reported diseases (1997–2019).

Characteristics	Selected Items from the List of (sd-PF) Diseases				Total
Count	1979	4085	833	489	7386
Arithmetic mean	86.04	177.61	36.22	23.68	74.83
Deviation	14.31	33.34	31.13	15.22	64.83
Maximum	156	261	80	52	238
Minimum	40	122	17	6	222

**Table 3:** Overview of indicators and their subcategories.

Indicator	Label	Subcategory
Sex	M	Male
	F	Female
Age category	Age1	Up to 29 years
	Age2	From 30 to 39 years
	Age3	40 to 49 years
	Age4	50 to 59 years
	Age5	60 and over
Economic activity sector	Sector 1	Agriculture and Forestry
	Sector 2	Mining and Quarrying
	Sector 3	Industrial Production
	Sector 4	Construction
	Sector 5	Others
Region of the medical facility	BL	Bratislava Region
	TA	Trnava Region
	NI	Nitra Region
	TC	Trenčín Region
	ZI	Žilina Region
	BC	Banská Bystrica Region
	PV	Prešov Region
	KI	Košice Region

**Table 4:** Overview of the number of (sd-PF) relative to the sex of workers (1997–2019).

	Men	Women	Total
Item 28			
Number	1934	45	1979
Arithmetic mean	84.09	1.96	43.02
Share (%)	97.73	2.27	100
Item 29			
Number	2241	1844	4085
Arithmetic mean	97.43	80.17	88.80
Share (%)	54.86	45.14	100
Item 38			
Number	783	50	833
Arithmetic mean	34.04	2.17	1.09
Share (%)	94.00	6.00	100
Items 33–44			
Number	471	18	489
Arithmetic mean	20.48	0.78	10.63
Share (%)	93.62	6.38	100
Total	5429	1957	7386
Share (%)	73.50	26.50	100

**Table 5:** Overview of (sd-PF) numbers by workers’ ages (1997–2019).

	To 29	30–39	40–49	50–59	Over-60
Item 28					
Number	29	326	845	723	56
Arithmetic mean	1.26	14.17	36.74	31.43	2.43
Share (%)	1.5	16.5	42.7	36.5	2.8
Item 29					
Number	31	440	1758	1760	96
Arithmetic mean	1.35	19.13	76.43	76.52	4.17
Share (%)	0.8	10.8	43.0	43.1	2.4
Item 38					
Number	1	19	194	476	143
Arithmetic mean	0.04	0.83	8.43	20.70	6.22
Share (%)	0.1	2.3	23.3	57.1	17.2
Items 33–44					
Number	0	20	74	145	250
Arithmetic mean	0.0	0.87	3.22	6.30	10.87
Share (%)	0.0	4.1	15.1	29.7	51.1
Total	61	805	2871	3104	545
Share (%)	0.8	10.9	38.9	42.0	7.4

**Table 6:** Overview of the numbers of (sd-PF) by sectoral economic activity (1997–2019).

	Sector 1	Sector 2	Sector 3	Sector 4	Sector 5
Item 28					
Number	562	896	328	110	83
Arithmetic mean	24.43	38.96	14.26	4.78	3.61
Share (%)	28.4	45.3	16.6	5.6	4.2
Item 29					
Number	324	1125	2221	171	244
Arithmetic mean	14.09	48.91	96.57	7.43	10.61
Share (%)	7.9	27.5	54.4	4.2	6.0
Item 38					
Number	66	169	479	50	69
Arithmetic mean	2.87	7.35	20.83	2.17	3.00
Share (%)	7.9	20.3	57.5	6.0	8.3
Items 33–44					
Number	2	329	123	22	13
Arithmetic mean	0.09	14.30	5.35	0.96	0.57
Share (%)	0.4	67.3	25.2	4.5	2.7
Total	954	2519	3151	353	409
Share (%)	12.9	34.1	42.7	4.8	5.5

**Table 7:** Overview of number of (sd-PF) by region of Slovakia (1997–2019).

	BL	TA	NI	TC	ZI	BC	PV	KI
Item 28								
Number	50	1	4	292	283	540	51	758
Arithmetic mean	2.17	0.04	0.17	11.70	12.30	23.48	2.22	32.96
Share (%)	2.5	0.1	0.2	14.8	14.3	27.3	2.6	38.3
Item 29								
Number	258	7	10	812	1067	763	58	1110
Arithmetic mean	11.22	0.30	0.43	35.30	46.39	33.17	2.52	48.26
Share (%)	6.3	0.2	0.2	19.9	26.1	18.7	1.4	27.2
Item 38								
Number	20	6	1	100	118	126	27	435
Arithmetic mean	0.87	0.26	0.04	4.35	5.13	5.48	1.17	18.91
Share (%)	2.4	0.7	0.1	12.0	14.2	15.1	3.2	52.2
Items 33–44								
Number	18	0	38	102	56	98	1	176
Arithmetic mean	0.78	0.00	1.65	4.43	2.43	4.30	0.04	7.65
Share (%)	3.7	0.0	7.8	20.9	11.5	20.0	0.2	36.0
Total	346	14	53	1306	1524	1527	137	2479
Share (%)	4.7	0.2	0.7	17.7	20.6	20.7	1.9	33.6

BL—Bratislava region, TA—Trnava region, NI—Nitra region, TC—Trenčín region, ZI—Žilina region, BC—Banská Bystrica region, PV—Prešov region, KI—Košice region.

**Table 8:** Overview of variables for comparing regions of Slovakia.

Label	Description
A1	Area of region (km^2^)
A2	Population of region (number)
A3	Working-age population of region (number)
A4	Average age in region (year)
A5	Unemployment rate in the region (%)
A6	Population working in Sector 1 region (number)
A7	Population working in Sector 2 region (number)
A8	Population working in Sector 3 region (number)
A9	Population working in Sector 4 region (number)
A10	Population working in Sector 5 region (number)
A11	Number of reported cases of selected occupational diseases (number) in the region—Item 28, Item 29, Item 38, Items 33–34

**Table 9:** Regional statistics for selected indicators.

Label	Description							
	BL	TA	NI	TC	ZI	BC	PV	KI
A1	2053 km^2^	4146 km^2^	6344 km^2^	4502 km^2^	6809 km^2^	9454 km^2^	8973 km^2^	6754 km^2^
A2	646,599	561,723	679,657	163,831	690,916	650,503	822,993	798,579
A3	436,199	391,962	471,261	407,039	481,203	448,751	565,187	547,611
	M: 212,184 F: 224,015	M: 198,035 F: 193,926	M: 238,273 F: 232,987	M: 206,839 F: 200,200	M: 244,115 F: 237,088	M: 225,987 F: 222,764	M: 287,704 F: 277,482	M: 275,799 F: 271,862
A4	40.97	41.99	42.77	42.78	40.12	41.98	38.85	39.77
A5	4%	7.02%	7.02%	4.62%	7.12%	11.64%	12.94%	10.74%
A6	1990	5094	7056	3736	4414	5486	5482	3833
A7	646	354	178	3299	613	597	295	564
A8	49,866	49,015	52,534	71,322	55,489	36,168	41,428	39,247
A9	14,034	5911	6904	5089	9605	6046	9593	7091
A10	312,740	86,023	108,830	85,819	113,723	103,229	108,259	128,208
A11	22	1	5	12	63	49	5	117

A1—Area of region, A2—Population of region, A3—Working-age population of region, A4—Average age in region, A5—Unemployment rate in the region, A6—Population working in Sector 1 region, A7—Population working in Sector 2 region, A8—Population working in Sector 3 region, A9—Population working in Sector 4 region, A10—Population working in Sector 5 region, A11—Number of reported cases of selected occupational diseases (number) in the region, BL—Bratislava region, TA—Trnava region, NI—Nitra region, TC—Trenčín region, ZI—Žilina region, BC—Banská Bystrica region, PV—Prešov region, KI—Košice region, M—Male, F—Female.

**Table 10:** Correlation matrix.

	A1	A2	A3	A4	A5	A6	A7	A8	A9	A10	A11
**A1**	1.00										
**A2**	0.57	1.00									
**A3**	−0.64	−0.05	1.00								
**A4**	−0.33	−0.84	−0.12	1.00							
**A5**	0.89	0.70	−0.51	−0.57	1.00						
**A6**	0.62	0.12	−0.72	0.22	0.46	1.00					
**A7**	−0.29	−0.41	−0.06	0.41	−0.45	−0.36	1.00				
**A8**	−0.54	−0.54	0.04	0.52	−0.78	−0.21	0.77	1.00			
**A9**	−0.34	0.27	0.86	−0.49	0.21	−0.54	−0.34	−0.14	1.00		
**A10**	−0.58	0.01	0.99	−0.18	−0.42	−0.70	−0.16	−0.08	0.87	1.00	
**A11**	0.26	0.46	−0.02	−0.40	0.32	−0.28	−0.12	−0.38	−0.01	0.02	1.00

A1—Area of region, A2—Population of region, A3—Working-age population of region, A4—Average age in region, A5—Unemployment rate in the region, A6—Population working in Sector 1 region, A7—Population working in Sector 2 region, A8—Population working in Sector 3 region, A9—Population working in Sector 4 region, A10—Population working in Sector 5 region, A11—Number of reported cases of selected occupational diseases (number) in the region.

**Table 11:** Summary of principal components analysis.

Components	Dim1	Dim2	Dim3	Dim4	Dim5	Dim6	Dim7
Eigenvalue	4.55	3.65	1.29	0.74	0.35	0.28	0.14
Variance (%)	41.37	33.17	11.72	6.68	3.21	2.57	1.28
Cumulative variance (%)	41.37	74.54	86.26	92.94	96.15	98.72	100.00

**Table 12:** Component matrix for the first three principal components.

	A1	A2	A3	A4	A5	A6	A7	A8	A9	A10	A11
Dim1	0.92	0.63	−0.71	−0.42	0.94	0.67	−0.46	−0.65	−0.40	−0.63	0.32
Dim2	−0.04	0.61	0.67	−0.76	0.22	−0.48	−0.52	−0.56	0.84	0.73	0.39
Dim3	0.05	0.21	−0.11	−0.24	0.03	−0.49	0.61	0.24	−0.20	−0.15	0.66

## Data Availability

Publicly available datasets were analyzed in this study. These data can be found here: http://www.nczisk.sk/en/Publications/Edition_Health_Statistics/Pages/Archive.aspx and https://slovak.statistics.sk/ (accessed on 28 August 2021).

## References

[1] Brhel P. (2003). Occupational Respiratory Diseases in the Czech Republic. Ind. Health.

[2] Kudász F., Nagy K., Nagy I. (2017). Occupational diseases in Belgium, the Czech Republic and Hungary—A Comparison. Cent. Eur. J. Occup. Environ. Med..

[3] Gómez M.G., López R.C., Ortiz Z.H., Soria F.S. (2017). Differences in the Recognition of Occupational Diseases by Sex, Occupation and Business Activity in Spain (1990–2009). Rev. Esp. Salud Publica.

[4] Świątkowska B., Szeszenia-Dąbrowska N. (2017). Long-term Epidemiological Observation of Asbestos-related Diseases in Poland, 1970–2015. Occup. Med..

[5] Oksa P., Sauni R., Talola N., Virtanen S., Nevalainen J., Saalo A., Uitti J. (2019). Trends in Occupational Diseases in Finland, 1975–2013: A Register Study. BMJ Open.

[6] Das B. (2020). Prevalence of Work-related Occupational Injuries and its Risk Factors Among Brickfield Workers in West Bengal, India. Int. J. Ind. Ergon..

[7] Boden L.I. (2020). The Occupational Safety and Health Administration at 50-the Failure to Improve Workers’ Compensation. Am. J. Public Health.

[8] Rushton L., Hutchings S.J., Fortunato L., Young C., Evans G.S., Brown T. (2012). Occupational Cancer Burden in Great Britain. Br. J. Cancer.

[9] Wang D., Liu A., Zhang S., Yu Y., Hu W., Sun X. (2020). History of the Development of the Reporting System of Occupational Diseases and Occupational Disease List in China. China CDC Wkly..

[10] Carder M., Bensefa-Colas L., Mattioli S., Noone P., Stikova E., Valenty M., Telle-Lamberton M. (2015). A review of Occupational Disease Surveillance Systems in Modernet Countries. Occup. Med..

[11] Nicholson P.J. (2002). Occupational Health in the European Union. Occup. Med..

[12] Rushton L. (2017). The Global Burden of Occupational Disease. Curr. Environ. Health Rep..

[13] Howard J. (2017). Occupational health issues in the USA. Occup. Med..

[14] Moyo D., Zungu M., Kgalamono S., Mwila C.D. (2015). Review of Occupational Health and Safety Organization in Expanding Economies: The Case of Southern Africa. Ann. Glob. Health.

[15] Piňosová M., Andrejiova M., Badida M., Moravec M. (2021). Occupational disease as the bane of workers’ lives: A chronological review of the literature and study of its development in slovakia. part 1. Int. J. Environ. Res. Public Health.

[16] Fosbroke J. (1831). Practical Observations on the Pathology and Treatment of Deafness. Lancet.

[17] Fosbroke J. (1831). Practical Observations on the Pathology and Treatment of Deafness. No. II. Lancet.

[18] Fosbroke J. (1831). Practical Observations on the Pathology and Treatment of Deafness. No. III. Lancet.

[19] Sabine P.E. (1932). Acoustics and Architecture.

[20] Fink D. (2019). A new definition of noise: Noise is unwanted and/or harmful sound. Noise is the new ‘secondhand smoke’. Proc. Meet. Acoust..

[21] Jerger J., Jerger S., Pepe P., Miller R. (1986). Race Difference in Susceptibility to Noise-Induced Hearing Loss. Am. J. Otol..

[22] Helzner E.P., Cauley J.A., Pratt S.R., Wisniewski S.R., Zmuda J.M., Talbott E.O. (2005). Race and Sex Differences in Age-related Hearing Loss: The Health, Aging and Body Composition Study. J. Am. Geriatr. Soc..

[23] Kyoko N., Nakao M. (2005). Effect of Smoking on Hearing Loss: Quality Assessment and Meta-analysis. Prev. Med..

[24] Hu H., Sasak N., Ogasawara T., Nagahama S., Akter S., Kuwahara K. (2019). Smoking, Smoking Cessation, and the Risk of Hearing Loss: Japan Epidemiology Collaboration on Occupational Health Study. Nicotine Tob. Res..

[25] Shlomo S., Gelfand A.S. (1981). The Relationship Between Magnitude of Hearing Loss and Acoustic Reflex Threshold Levels. J. Speech Hear. Disord..

[26] Humes L.E. (1984). Noise-induced Hearing Loss as Influenced by other Agents and by Some Physical Characteristics of the Individual. J. Acoust. Soc. Am..

[27] Rabinowitz P.M. (2000). Noise-Induced Hearing Loss. Am. Fam. Physician.

[28] Agarwal S., Mishra A., Jagade M., Kasbekar V., Nagle S.K. (2013). Effects of Hypertension on Hearing. Indian J. Otolaryngol. Head Neck Surg..

[29] Piňosová M., Andrejiová M., Lumnitzer E. (2018). Synergistic effect of risk factors and work environmental quality. Qual.-Access to Success.

[30] Moravec M., Liptai P., Dzuro T., Badida M. (2017). Design and Effectiveness Verification of Sound Reduction Measures in Production Hall. Adv. Sci. Technol. Res. J..

[31] Moravec M., Badida M., Mikusova N., Sobotova L., Svajlenka J., Dzuro T. (2021). Proposed Options for Noise Reduction from a Wastewater Treatment Plant: Case Study. Sustainability.

[32] Pyykkö I., Starck J., Färkkilä M., Hoikkala M., Korhonen O., Nurminen M. (1981). Hand-arm Vibration in the Aetiology of Hearing Loss in Lumberjacks. Br. J. Ind. Med..

[33] Iki M., Kurumatani N., Hirata K., Moriyama T., Satoh M., Arai T. (1986). Association between Vibration-Induced white Finger and Hearing Loss in Forestry Workers. Scand. J. Work. Environ. Health.

[34] Iki M. (1994). Vibration-induced White Finger as a Risk Factor for Hearing Loss and Postural Instability. Nagoya J. Med. Sci..

[35] Bovenzi M. (2006). Health Risks from Occupational Exposures to Mechanical Vibration. Med. Lav..

[36] House R.A., Sauvé J.T., Jiang D. (2010). Noise-induced Hearing Loss in Construction Workers Being Assessed for Hand-arm Vibration Syndrome. Can. J. Public Health.

[37] Pettersson H., Burström L., Hagberg M., Lundström R., Nilsson T. (2014). Risk of Hearing Loss among Workers with Vibration-Induced White Fingers. Am. J. Ind. Med..

[38] Turcot A., Girard S.A., Courteau M., Baril J., Larocque R. (2015). Noise-induced hearing loss and combined noise and vibration exposure. Occup. Med..

[39] World Health Organization 1 in 4 People Projected to Have Hearing Problems by 2050. https://www.who.int/news/item/02-03-2021-who-1-in-4-people-projected-to-have-hearing-problems-by-2050.

[40] Health and Safety Executive Noise at Work. https://www.hse.gov.uk/noise/index.htm.

[41] Chen K.-H., Su S.-B., Chen K.-T. (2020). An overview of occupational noise-induced hearing loss among workers: Epidemiology, pathogenesis, and preventive measures. Environ. Health Prev. Med..

[42] Raynaud M. (1862). De L’asphyxie Locale et de la Gangrène Symétrique des Extrémités.

[43] Loriga G. (1911). Il Lavoro Con i Martelli Pneumatici. Boll Inspett Lav..

[44] Eger T., Thompson A., Leduc M., Krajnak K., Katie Goggins A.G. (2014). Vibration Induced White-Feet: Overview and Field Study of Vibration Exposure and Reported Symptoms in Workers. Work.

[45] Maheľová L., Dostálová K., Bátora I., Bízik A., Kukučková L., Moricová Š. (2012). Raynaudov fenomén ako súčasť choroby z vibrácií. Via Pract..

[46] Gemne G., Pyykkö I., Taylor W., Pelmear P.L. (1987). The Stockholm Workshop Scale for the Classification of Cold-induced Raynaud’s Phenomenon in the Hand-arm Vibration Syndrome. Scand. J. Work. Environ. Health.

[47] Sauni R., Virtema P., Pääkkönen R., Toppila E., Pyykkö I., Uitti J. (2010). Quality of Life (EQ-5D) and Hand-arm Vibration Syndrome. Int. Arch. Occup. Environ. Health.

[48] Sauni R., Pääkkönen R., Virtema P., Jäntti V., Kähönen M., Toppila E. (2009). Vibration-induced White Finger Syndrome and Carpal Tunnel Syndrome among Finnish Metal Workers. Int. Arch. Occup. Environ. Health.

[49] Milosavljevic S., Bagheri N., Vasiljev R.M., Mcbride D.I., Rehn B. (2012). Does Daily Exposure to Whole-Body Vibration and Mechanical Shock Relate to the Prevalence of Low Back and Neck Pain in a Rural Workforce?. Ann. Occup. Hyg..

[50] McBride D., Paulin S., Herbison G.P., Waite D., Bagheri N. (2014). Low Back and Neck Pain in Locomotive Engineers Exposed to Whole-Body Vibration. Arch. Environ. Occup. Health.

[51] Kubo M., Terauchi F., Aoki H., Matsuoka Y. (2001). An investigation into a synthetic vibration model for humans: An investigation into a mechanical vibration human model constructed according to the relations between the physical, psychological and physiological reactions of humans exposed to vibration. Int. J. Ind. Ergon..

[52] Gerhardsson L., Ahlstrand C., Ersson P., Gustafsson E. (2020). Vibration-induced injuries in workers exposed to transient and high frequency vibrations. J. Occup. Med. Toxicol..

[53] Coelho D.A. (2020). Social, cultural and working conditions determinants of fatal and non-fatal occupational accidents in Europe. Sigurnost.

[54] Parent-Thirion A., Biletta I., Cabrita J., Vargas Llave O., Vermeylen G., Wilczynska A., Wilkens M. (2017). European Foundation for the Improvement of Living and Working Conditions. 6th European Working Conditions Survey.

[55] Krajnak K. (2018). Health effects associated with occupational exposure to hand-arm or whole body vibration. J. Toxicol. Environ. Health B Crit. Rev..

[56] Health and Safety Executive Cracks Down Hard on Dust. https://press.hse.gov.uk/2021.

[57] Silica, Crystalline. https://www.osha.gov/silica-crystalline.

[58] Beer C., Kolstad H.A., Søndergaard K., Bendstrup E., Heederik D., Olsen K.E., Omland Ø., Petsonk E., Sigsgaard T., Sherson D.L. (2017). A systematic Review of Occupational Exposure to Coal Dust and the Risk of Interstitial Lung Diseases. Eur. Clin. Respir. J..

[59] IEA World Total Coal Production, 1971–2019 Provisional. https://www.iea.org/data-and-statistics/charts/world-total-coal-production-1971-2019-provisional.

[60] Ramazzini B. (2000). De Morbis Artificum Diatriba 1700.

[61] King T.E. Hypersensitivity Pneumonitis (Extrinsic Allergic Alveolitis): Epidemiology, Causes, and Pathogenesis. https://www.uptodate.com/contents/hypersensitivity-pneumonitis-extrinsic-allergic-alveolitis-epidemiology-causes-and-pathogenesis?search=Hypersensitivity%20Pneumonitis%20(Extrinsic%20Allergic%20Alveolitis):%20Epidemiology,%20Causes,%20and%20Pathogenesis&source=search_result&selectedTitle=1~107&usage_type=default&display_rank=1.

[62] King T.E., Nicholson A. Hypersensitivity Pneumonitis (Extrinsic Allergic Alveolitis): Clinical manifestations and Diagnosis. https://www.uptodate.com/contents/hypersensitivity-pneumonitis-extrinsic-allergic-alveolitis-clinical-manifestations-and-diagnosis?search=Hypersensitivity%20Pneumonitis%20(Extrinsic%20Allergic%20Alveolitis):%20Clinical%20manifestations%20and%20Diagnosis&source=search_result&selectedTitle=1~107&usage_type=default&display_rank=1.

[63] Malo J.-L., Cartier A., Desjardins A., Weyer R.V., Vandenplas O. (1995). Occupational Asthma Caused by oak Wood Dust. Chest.

[64] Pérez-Ríos M., Ruano-Ravina A., Etminan M., Takkouche B. (2010). A Meta-analysis on Wood Dust Exposure and Risk of Asthma. Allergy.

[65] Enarson D.A., Chan-Yeung M. (1990). Characterization of Health Effects of Wood Dust Exposures.

[66] Seifert S.A., Essen S.V., Jacobitz K., Crouch R., Lintner C.P. (2003). Organic Dust Toxic Syndrome: A Review. J. Toxicol. Clin. Toxicol..

[67] Oxman A.D., Muir D.C.F., Shannon H.S., Stock S.R., Hnizdo E., Lange H.J. (1993). Occupational Dust Exposure and Chronic Obstructive Pulmonary Disease: A Systematic Overview of the Evidence. Am. Rev. Respir. Dis..

[68] Coggon D., Taylor A.N. (1998). Coal Mining and Chronic Obstructive Pulmonary Disease: A Review of the Evidence. Thorax.

[69] Ozga A.M., Obuchowska A., Standyło A., Wójcik J., Obuchowska K. (2020). Wood Dust Exposure and Risk of Sinonasal Cancer Development. J. Educ. Health Sport.

[70] Acheson E.D., Cowdell R.H., Hadfield E., Macbeth R.G. (1968). Nasal Cancer in Woodworkers in the Furniture Industry. Br. Med. J..

[71] Luttmann A., Jäger M., Griefahn B., Caffier G., Liebers F., Steinberg U. (2003). Preventing Musculoskeletal Disorders in the Workplace.

[72] Healthy Workplaces Campaign 2020–22. https://healthy-workplaces.eu/.

[73] U.S. Bureau of Labor Statistics Occupational Injuries and Illnesses Resulting in Musculoskeletal Disorder, May 2020. https://www.bls.gov/iif/oshwc/case/msds.htm.

[74] Cieza A., Causey K., Kamenov K., Hanson S.W., Chatterji S., Vos T. (2020). Global estimates of the need for rehabilitation based on the Global Burden of Disease study 2019: A systematic analysis for the Global Burden of Disease Study 2019. Lancet.

[75] Jolliffe I.T. (2002). Principal Component Analysis and Factor Analysis, Chap 7. Principal Component Analysis.

[76] Everitt B.S., Landau S., Leese M., Daniel S. (2011). Cluster Analysis.

[77] Hartigan J.A. (1975). Clustering Algorithms.

